# An *in vitro* assessment of liposomal topotecan simulating metronomic chemotherapy in combination with radiation in tumor-endothelial spheroids

**DOI:** 10.1038/srep15236

**Published:** 2015-10-15

**Authors:** Amar Jyoti, Kyle D. Fugit, Pallavi Sethi, Ronald C. McGarry, Bradley D. Anderson, Meenakshi Upreti

**Affiliations:** 1 Department of Pharmaceutical Sciences, University of Kentucky, Lexington, KY; 2 Department of Radiation Medicine, University of Kentucky Chandler Hospital, Lexington, KY

## Abstract

Low dose metronomic chemotherapy (LDMC) refers to prolonged administration of low dose chemotherapy designed to minimize toxicity and target the tumor endothelium, causing tumor growth inhibition. Topotecan (TPT) when administered at its maximum tolerated dose (MTD) is often associated with systemic hematological toxicities. Liposomal encapsulation of TPT enhances efficacy by shielding it from systemic clearance, allowing greater uptake and extended tissue exposure in tumors. Extended release of TPT from liposomal formulations also has the potential to mimic metronomic therapies with fewer treatments. Here we investigate potential toxicities of equivalent doses of free and actively loaded liposomal TPT (LTPT) and compare them to a fractionated low dose regimen of free TPT in tumor-endothelial spheroids (TES) with/without radiation exposure for a prolonged period of 10 days. Using confocal microscopy, TPT fluorescence was monitored to determine the accumulation of drug within TES. These studies showed TES, being more reflective of the *in vivo* tumor microenvironment, were more sensitive to LTPT in comparison to free TPT with radiation. More importantly, the response of TES to low-dose metronomic TPT with radiation was comparable to similar treatment with LTPT. This TES study suggests nanoparticle formulations designed for extended release of drug can simulate LDMC *in vivo*.

Traditional chemotherapeutic regimens incorporate the maximum tolerated dose (MTD)^1^ resulting in systemic toxicity. These toxicities mandate rest periods between cycles of therapy and result in re-growth of drug-resistant tumor cells and growth of more malignant tumors with no therapeutic response^2^. Low dose metronomic chemotherapy (LDMC) designed to minimize toxicity, functions by targeting the tumor microenvironment, particularly the endothelial cells, by frequent, fractionated doses of MTD. The potential need for frequent administrations has not allowed LDMC to advance beyond investigational applications. Topotecan (TPT), a water soluble camptothecin with topoisomerase I inhibitory activity when administered at its maximum tolerated dose (MTD) is often associated with systemic hematological toxicities. Metronomic oral TPT has been reported to have beneficial outcomes in clinical trials^3^,^4^ and preclinical tumor models^5^,^6^ of various cancer types. Liposomal encapsulation of TPT enhances its efficacy by shielding it from systemic clearance, allowing greater uptake and extended tissue exposure in solid tumors^7^,^8^. Extended release of TPT from liposomal formulations also has the potential to mimic metronomic therapies with fewer treatments. Endothelial cells in tumors are typically more sensitive than other tumor cells to prolonged LDMC^9^. Targeting these endothelial cells blocks tumor angiogenesis and eventually inhibits tumor growth^10^,^11^. However, similar to other anticancer therapies, inherent resistance to LDMC is also frequent^12^. Existing pre-clinical tumor cell models have yet to incorporate the variables of angiogenesis and intercellular heterogeneity of the tumor microenvironment. Furthermore, evaluating LDMC before extending it to preclinical animal models and clinical settings has also been difficult due to the above mentioned variables^13^.

The inadequacies of the tumor microenvironment in most *in vitro* tumor cell models has also made the design of drug delivery systems with optimal drug release rates challenging. The chronically aberrant vascular pathophysiology of developing tumors results in a leaky vasculature and poor lymphatic drainage^14^ that allows nanosized drug carriers like liposomes to preferentially accumulate. The enhanced permeability and retention (EPR) effect can result in 10–50 fold higher concentration of nanoparticles in tumors than in normal tissue^15^. Furthermore, sustained release of active drug from these nanoformulations simulates the action of metronomic therapy^16^. However, accumulation of drug-loaded liposomes in tumors as a consequence of the EPR effect may not lead to optimal free drug concentrations at the tumor site, and thus may not be a sufficient indicator of therapeutic efficacy^17^. Traditional 2D monolayer cell culture systems do not exemplify the complexity of the tumor and its microenvironment and are also not optimal for assessing nanoparticle efficacy^18^. Therefore, an *in vitro* tumor model that is amenable to monitoring nanoparticle penetration, drug release, and their subsequent effects on tumor cell viability and/or tumor growth is needed. The 3D system of tumor and endothelial cells co-cultured in ‘hanging drops’ recreates aspects of the tumor microenvironment, offering a simple yet potentially powerful *in vitro* tumor model to study therapeutic response. These avascular multicellular TES recreate the gradient of hypoxia, pH and interstitial pressure that results in the inner necrotic and the outer proliferating layer of cells similar to solid tumors *in vivo*^19^,^20^. The co-culture of different cell types in 3D also facilitates the inter-cellular crosstalk leading to the formation of an extracellular matrix known to influence the therapeutic response in cancer^21^. Liposomal formulations of TPT enhance the anticancer efficacy of TPT and significantly reduce tumor growth^7^,^8^ but reasons behind this outcome are not fully understood. In the current study, a TES model of triple negative breast cancer (TNBC) was used to investigate whether the extended release and drug penetration from liposomal TPT provided similar cytotoxicity as metronomic dosing.

Radiation alters the adjacent tumor microenvironment often with consequences for cancer cells beyond the direct effects of the radiation itself 22. Radiation therapy with concomitant or delayed chemotherapy is given to almost 70% of the patients with cancer. TPT has also been shown to induce radiosensitization upon prior or concurrent treatment with radiation^23^. Therefore, this study investigates the combined effect of a clinically relevant dose of radiation (3 Gy) in conjunction with free or liposomal on avascular tumor-endothelial spheroids over an extended period of 10 days.

## Results

### Extended release kinetics of LTPT in physiological buffer and culture medium

**Figure 1:**
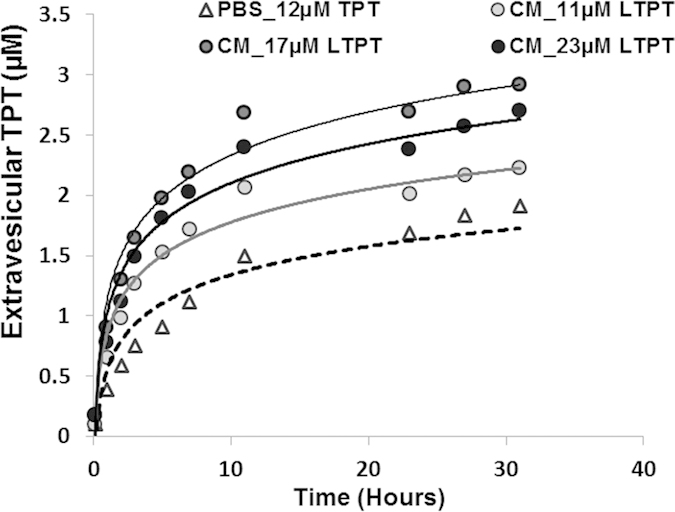
Extravesicular TPT concentrations versus time in LTPT suspensions at various dilutions in PBS or culture media at pH 7.4 and 37 °C. Release profile of LTPT formulations in PBS buffer and spheroid culture media at pH 7.4 and 37 °C. Graph represents the extravesicular TPT concentration calculated over time in the TES culture media and PBS buffer. The extravesicular TPT was quantified by monitoring TPT fluorescence at ex. and em. wavelengths of 410 and 550 nm, respectively. The lines are trendlines for the four different release experiments.

Simulating metronomic dosing requires sustained release of TPT from liposomes under study conditions. Using a spectroscopic method developed in previous work to monitor the release kinetics of LTPT in aqueous buffer and biological fluids^24^, TPT release was monitored in cell culture medium to determine if LTPT formulations provided sustained release in TES media. Release kinetics of three different LTPT suspensions (11, 17 and 23 μM total suspension TPT) in TES media are shown in Fig. 1 and were compared to release from LTPT (12 μM total suspension TPT) in pH 7.4 PBS. While slightly greater in cell culture media than in PBS, TPT release was still sustained over the 31 hr period release was monitored. This time frame is relevant when considering TESs treated with LTPT were washed after 24 hr of exposure to the formulation.

### Higher intrinsic sensitivity of endothelial cells to TPT alone and in combination with radiation than tumor cells

**Figure 2:**
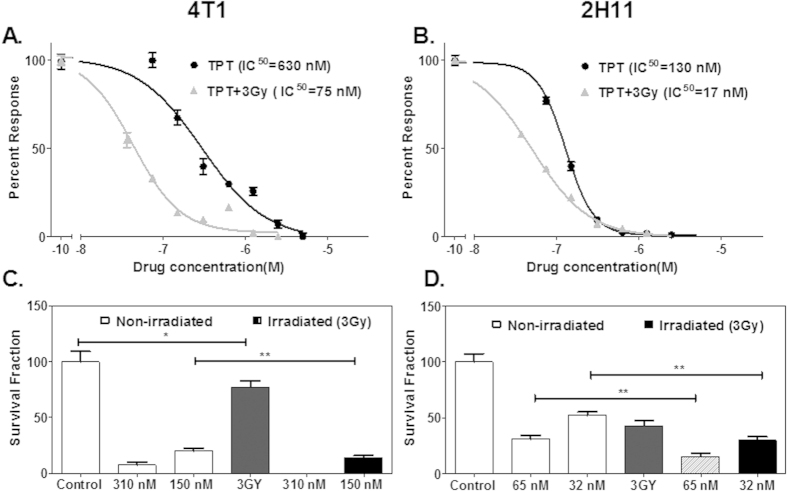
Endothelial cells are more sensitive to low concentrations of TPT alone and in combination with radiation in comparison to tumor cells. Effect of TPT treatment on the viability of tumor (**A**) and endothelial cells (**B**) was assessed with or without radiation (3 Gy) exposure in a 72h MTT assay. IC50 values obtained from the MTT assay indicate that X-ray irradiation post TPT treatment considerably decreases the viability of both tumor and endothelial cells. Long term survival studies further validate the impact of radiation (3y) on tumor and endothelial cell colony formation with and without TPT at ½ or ¼ of the IC50 concentrations. For the survival assay, cells were exposed to drug and/or radiation and then grown in drug-free medium for 10–14 days. Colonies (>50 cells) were stained with crystal violet and counted (Materials and Methods). Data represent means ± SD (n = 3). Significant changes were recorded as **p < 0.01; *p < 0.05.

Cellular viability was determined by MTT assay in monolayer cell cultures of 4T1 mammary breast carcinoma and 2H11 endothelial cell lines treated with increasing concentrations of TPT. Comparing Fig. 2A,B clearly demonstrates that endothelial cells are more sensitive to TPT treatment. The half maximal inhibitory concentrations (IC50) of TPT were 130 nM for endothelial cells and 630 nM for tumor cells. Combined treatment of TPT in conjunction with radiation further sensitized both the tumor and endothelial cells in the 72 hour MTT assay. An approximate 10-fold decrease was evident in the values of the IC50 of the tumor cells from 630 nM to 75 nM and endothelial cells from 130 to 17 nM, respectively, when TPT treatment was combined with 3 Gy of radiation after 6 hours.

Ionizing radiation triggers a myriad of responses in cells, including apoptosis, necrosis, stress-induced premature senescence (SIPS) and autophagy. Most of these responses result in loss of colony-forming ability that may be assessed in the clonogenic survival assay^25^. Clonogenic studies to understand the effects of TPT and radiation exposure of 3 Gy in tumor and endothelial cell lines further indicated that TPT concentrations at one-half and one-fourth of the IC50 were more effective in inhibiting survival of both cell types (Fig. 2C,D).

### Tumor-endothelial cell spheroids (TES): an *in vitro* model to assess drug penetration and accumulation

**Figure 3:**
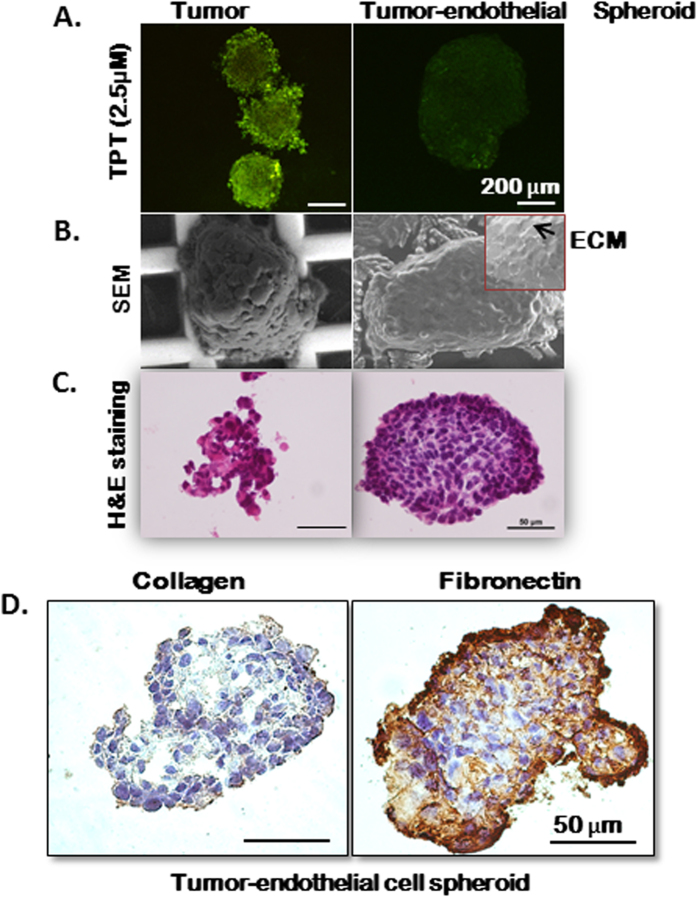
Tissue-like integrated morphology and development of extracellular matrix prevents penetration of TPT in the TES. (**A**) Tumor cell only and tumor-endothelial cell (TES) spheroids were incubated in 2.5 μM TPT for 6 hours and extent of uptake was assessed using fluorescent confocal imaging. Efficient uptake of TPT imaged at excitation wavelength 410 nm was observed in tumor cell spheroids. There was minimal penetration of the fluorescent TPT (green). Scanning electron micrographs (**B**), histology (**C**) and immunostaining for ECM proteins (**D**) like collagen and fibronectin indicate the compactness as well as the presence of ECM in the TES.

Tumors *in vivo* are not merely aggregates of cancer cells but are representative of structurally and functionally deregulated organs with a 3D tissue architecture contributed by multiple cell types and the extracellular matrix^26^,^27^. Gravity-enforced self-assembly generates multicellular scaffold-free aggregates in a self-contained microenvironment^28^. The penetration of free TPT was assessed at excitation wavelength of 410 nm in tumor cell only and tumor-endothelial cell spheroids (TES) incubated in 2.5 μM TPT for 6 hours by confocal fluorescence microscopy. The drug uptake within 6 hours of incubation was noticeably higher in the tumor cell only spheroids in comparison to tumor-endothelial cell spheroids (TES) (Fig. 3A). Scanning electron microscopy and H & E staining revealed that while tumor cells remain as aggregates and fail to form intact spheroids, the tumor and endothelial cells in the TES developed an integrated and compact architecture with indications of ECM formation (Fig. 3B,C). Immunohistochemical (IHC) studies indicated that the presence of both tumor and endothelial cells in 3-dimensions causes the production of extracellular matrix proteins like fibronectin and collagen. These factors may be responsible for the integrated tissue-like morphology and greatly limited penetration of TPT (Fig. 3D). The presence of extracellular matrix (ECM) has been reported to affect the penetration of therapeutic agents in solid tumors^29^ as observed in the present study. The tumor cell only spheroids exist as unattached cell aggregates and are either washed out during the IHC procedures or have minimal expression of ECM proteins (data not shown).

### Higher TES accumulation and penetration of TPT from liposomal formulations

**Figure 4:**
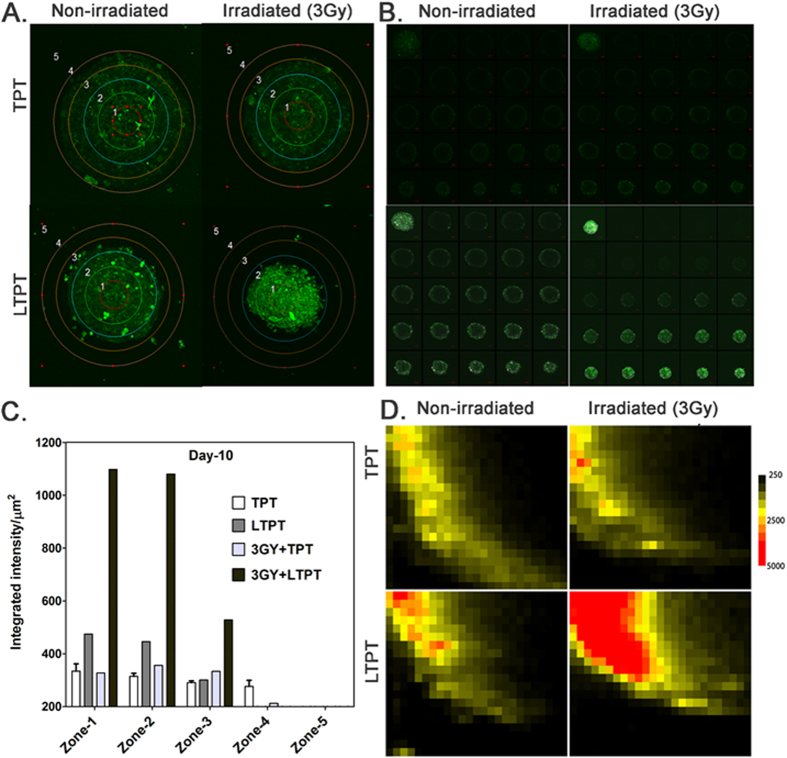
Liposomal TPT formulation facilitates penetration of TPT in TES over an extended period of 10 days. Comparisons of intra-spheroidal TPT uptake in TES when incubated in the presence of either free or liposomally entrapped TPT for 24 hours with and without radiation exposure of 3 Gy at day 10 post-treatment. (**A**) Confocal TES images were partitioned into 5 concentric zones for quantification of TPT accumulation (Zones (1–5). (**B**) Representative confocal images after treatment of 24 optical slices (i.e. each z-stack image) taken every 5 μm from the center to bottom of the intact TES. The top left image in each tile is a 3D reconstruction of the stack of 24 confocal optical sections. (**C**) Quantification of TPT accumulation based on the integrated TPT fluorescence intensity (integrated intensity normalized to surface area in each zone to avoid bias) in each zone of the TES after treatment with liposomal TPT. (**D**) Heat map of fluorescent confocal images showing the intracellular accumulation of TPT across the y and z-axis of TES. Each image represents averages of fluorescence intensity of 4 TES within the same treatment group.

The TES were incubated in 5 μM of free or LTPT with and without radiation exposure (3 Gy) for 24 hours. The media was replaced and the spheroids were assessed for accumulation and penetration of TPT at day 10. Sustained release of drug from liposomes and changes caused by radiation are expected to occur over a period of time *in vivo*. Therefore, drug accumulation and cytotoxicity in TES was assessed at 10 days post-treatment for optimal results. Maximum intensity projections of a stack of 24 confocal images at a 5 μm step size of each TES were portioned into 5 concentric zones as shown in Fig. 4A. The integrated intensity normalized to surface area of each respective zone in every treatment group was represented in Fig. 4C. The quantification of TPT accumulation based on the fluorescence intensity in each zone of the TES 10 days after treatment indicates significantly higher accumulation in zone-1 and zone -2 of TES incubated in LTPT following radiation exposure (Fig. 4C). Figure 4B is a representative image showing a confocal image of a TES in tile mode, with accumulation and penetration of the drug in the 24 optical sections at different z-levels. Heat maps depicting the distribution of TPT within the TES in different treatment groups further indicate that the drug penetration was more efficient in TES incubated in LTPT and exposed to a 3 Gy dose of radiation (see Fig. 4D). Absence of the outer zones (3, 4 and 5) reflects a decrease in TES size. Penetration of TPT to the inner layers of the TES is indicative of the higher level of cytotoxicity resulting from the combined treatment of LTPT and radiation in comparison to the other treatment groups.

Free and liposomal formulations of TPT at concentrations of 5 μM were also prepared in the pH 7.4 culture medium used to grow the TES. Six hours later, treated TES were imaged by confocal florescence microscopy at an excitation wavelength of 410 nm. The fluorescence intensity of the culture medium with liposomally entrapped TPT (LTPT) was exceptionally low in comparison to free TPT (Supplementary Figure. 1). Similar images were obtained at 24 hours, the time point when the TES medium was replaced with fresh medium **(data not shown)**. These results, in addition to those in Fig. 4, suggest that TPT-loaded liposomes accumulate in the TES within 24 hours, followed by extended release of the drug during the 10 day studies.

### Low concentrations of LTPT and radiation enhance TES cytotoxicity when assessed over a prolonged period of time

**Figure 5:**
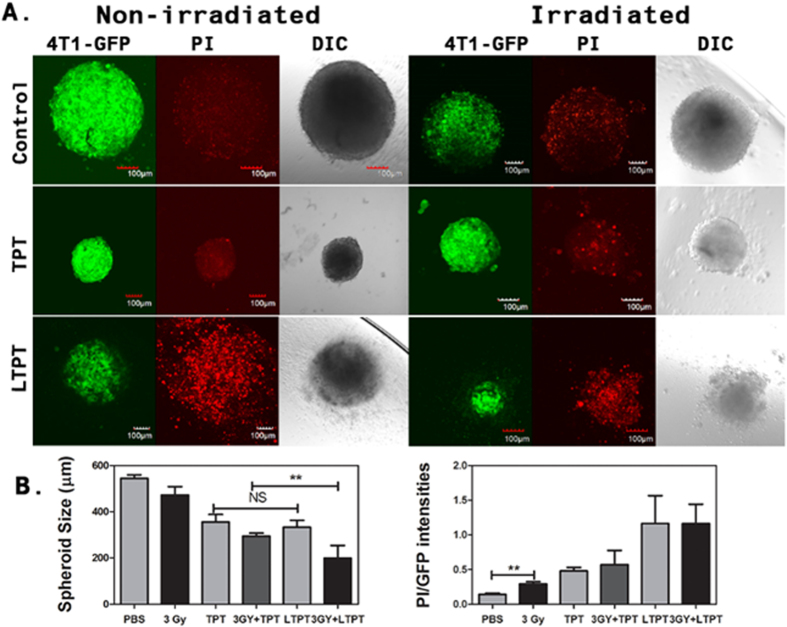
Enhanced cytotoxicity in TES upon combined treatment with a low concentration of liposomal TPT (LTPT) and radiation. (**A**) Representative confocal images of GFP expressing tumor cells and PI uptake elucidating the effect of free and entrapped TPT treatment (250 nM) with and without radiation (3 Gy). Each treatment was expected to reduce the overall fluorescence of GFP expressing 4T1 tumor cells, increase the accumulation of the PI stain and decrease the size of TES. These effects are indicative of the level of cytotoxicity conferred by each treatment. Increase in PI staining, decrease in spheroid size and GFP expression were observed in TES treated with LTPT followed by radiation exposure, indicating higher cytotoxicity. (**B**) Spheroid size and the ratio of PI/GFP intensity were evaluated using ImageJ software. (n = 12 TES for spheroid size per treatment and n = 4 TES for PI/GFP ratio) **P < 0.01.

The anti-tumor effects of radiation and TPT occur via their impact on the tumor microenvironment, particularly the endothelial cells^30^. The response of the tumor/tumor microenvironment to combined therapy using liposomally-entrapped TPT and radiation was assessed in 3D co-cultures of tumor and endothelial cells (TES) by incubating at a TPT concentration of 250 nM followed by exposure to a clinically relevant dose of radiation (3 Gy). The selected TPT concentration was 2-fold higher than the half maximal inhibitory concentration (IC50) for endothelial cells and 3-fold less than the IC50 for the tumor cells (Fig. 2). The effect of free and LTPT with and without radiation (3 Gy) was observed on day 10 by assessing the size, GFP expression of 4T1 tumor cells and extent of cell damage by Propidium Iodide (PI) staining of TES (Fig. 5A,B).

PI is a fluorescent nuclear and chromosome counterstain that is impermeant to live cells, making it a useful indicator of dead cells within a population. The emission maximum for the dye bound to DNA is 617 nm and therefore it fluoresces red^31^. TES subjected to radiation exposure (3 Gy) alone underwent a decrease in size at day 10 with a moderate increase in PI staining. While staining with PI was significant in TES that were incubated in LTPT with and without radiation exposure (3 Gy) the combined effects of a decrease in size and cell damage as observed by PI staining were significantly higher in TES that were subject to incubation with LTPT in conjunction with radiation (Fig. 5A). High throughput evaluations of spheroid size and ImageJ-based determinations of decrease in the fluorescence intensity of green fluorescent (GFP) expressing 4T1 tumor cells (green) and increase of PI staining in the TES further validate the enhanced cytotoxicity induced by LTPT in conjunction with radiation (Fig. 5B).

### Extended release of TPT from liposomes mimics the low dose metronomic dosing effect in TES

**Figure 6:**
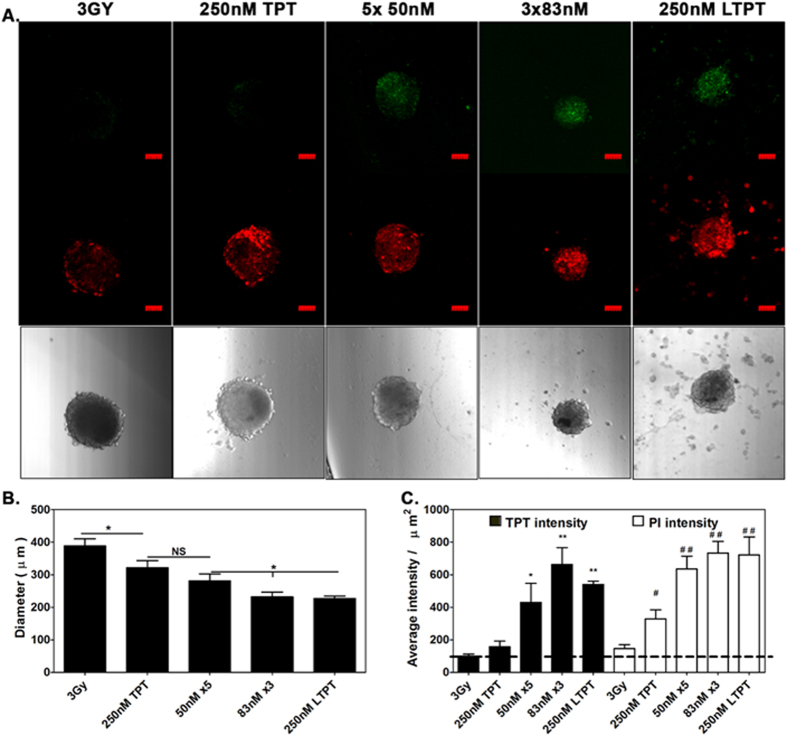
A single LTPT treatment results in TES toxicity levels that are comparable to the fractionated (metronomic) dosing regimen using free TPT in conjunction with radiation. The toxicity of repeated fractionated dosing was investigated against a single dose of free TPT or LTPT followed by a single exposure to radiation (3 Gy) in each treatment after the first dose. Fractionated regimens consisting of 3 × 83 nM or 5 × 50 nM TPT treatments with exchanges occurring each 24 hours were given and toxicity to TES was evaluated at day 10 post-treatment. (**A**) Fluorescence confocal images indicate greater uptake of PI (red) and accumulation of TPT (green) in the TES treated with repeated low concentrations of free TPT or the liposomal formulation in comparison to a single treatment of free TPT at a higher concentration (250 nM). Representative DIC images with decreases in the size of the TES confirm the associated cytoxicity in these treatment groups. (**B**) Quantitative analysis of spheroid size, TPT and PI intensity assessed at day 10 post-treatment for the various treatment groups described indicates that the effect of the liposomal formulation was comparable to the fractionated dosing of free TPT. This is supported by the lack of significance in in TPT or PI uptake between LTPT and the metronomic TPT dosing. (n = 12 TES for spheroid size per treatment and n = 4 TES for PI staining and GFP expression) *P < 0.05; **P < 0.01.

**Figure 7:**
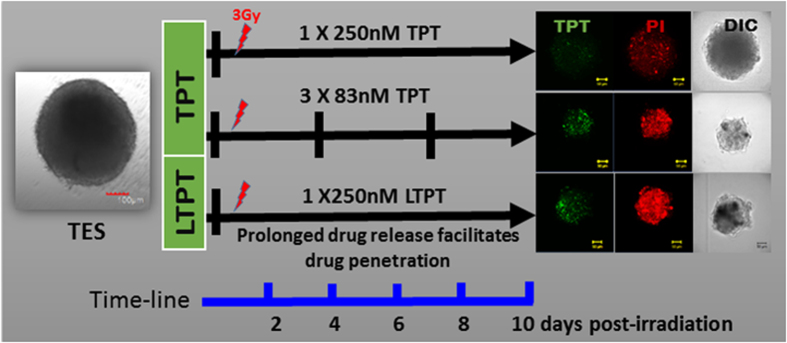
A schematic representation of free and liposomally entrapped TPT (LTPT) compared to the metronomic dosing regimen of free TPT studied in TES. Treatment response was determined by evaluating the changes in fluorescence intensity of TPT uptake, spheroid sizing of DIC (differential interference contrast) images, and propidium iodide (PI) staining of dead/damaged cells.

Metronomic chemotherapy targets the tumor microenvironment, particularly the endothelial cells which are more sensitive to lower and sustained doses of drugs than the tumor cells. Inhibiting tumor endothelial cells disrupts the normal interaction of tumor cells with their microenvironment, making it difficult for the tumor cells to survive and eventually the inhibition of tumor growth^10^. As shown in the present study and reported earlier, endothelial cell types are more sensitive to TPT than tumor cell types (Fig. 2 and^32^). The sustained release of liposomally entrapped TPT was expected to simulate low dose metronomic chemotherapy. To investigate whether the metronomic dosing effect can be replicated *in vitro*, the responses of TES to a single dose of free TPT, liposomally entrapped TPT, and fractionated low doses of TPT in conjunction with radiation exposure were compared. TES were treated with free or entrapped TPT (250 nM) followed by radiation exposure after 6 hours. The culture media was replaced after 24 hours as described earlier for toxicity evaluation. For low dose metronomic treatment, the single dose of free TPT was fractionated into 3 or 5 doses given every 24 hours. Thus, the treatments consisted of 83 nM concentrations of free TPT every 24 hours for 3 days (3 fractions, 3 × 83 nM) or 50 nM concentrations of TPT every 24 hours for 5 days (5 fractions, 5 × 50 nM) followed by subsequent replacement with fresh medium 24 hours after each treatment. At day10 the TES were imaged for sizing, drug uptake and cell damage by confocal fluorescence microscopy. Decrease in TES size correlated with increased accumulation of TPT (green) and enhanced cell damage observed by PI staining (Red). The treatment results in TES that received fractionated dosing and radiation were comparable to those treated with the single dose of LTPT followed by radiation exposure (3 Gy) (Fig. 6A). Of the two free TPT metronomic treatment regimens, the 3 × 83 nM regimen was more effective than the 5 × 50 nM regimen (Fig. 6B). The results further suggest the relevance of an optimal *in vitro* model for preclinical evaluation of LDMC. Figure 7 provides a schematic comparing free, liposomal and metronomic dosing regimen of TPT in conjunction with radiation on TES at 10 days post treatment.

### Enhanced cytotoxicity of LTPT with radiation results from differential intracellular phosphoMAPK signaling in the endothelial component of the TES

**Figure 8:**
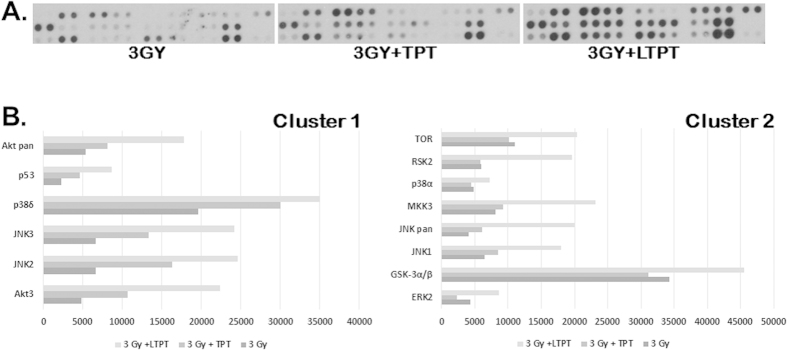
Phospho-MAP kinase expression profiling reveals elevated activation of proteins associated with stress and apoptotic signaling in TES treated with LTPT and radiation in comparison to a similar concentration of free TPT. (**A**) The human phosho-MAPK array was utilized to detect upregulation of several phosphoproteins in the TES spheroids treated with 250 nM LTPT or free TPT followed by radiation exposure of 3 Gy after 6 hours. TES subject to radiation exposure alone served as a control. Data shown are from a 60 second exposure of the array blot to x-ray film. (**B**) The graphs of pixel intensities (cluster 1 and 2) representative of phosphoprotein expression profiles in TES upon treatment with free TPT and liposomally entrapped nanoformulation (LTPT) combined with radiation exposure.

A semi-quantitative comparison of the expression of 26 phosphoproteins involved in the stress and apoptotic signaling pathway in response to free TPT and LTPT at day 10 was performed by probing the Phospho-MAPK antibody arrays with TES lysates (Fig. 8A). ImageJ analyses of pixel intensities was used to identify the differential expression of phosphoproteins under different treatment conditions. The heat map generated (Fig. 8B) revealed a 2–5 fold increase in phosphorylation of JNK and p38 and ERK family of MAP kinases in the TES treated with LTPT and radiation. JNK and p38 are stress activated MAP kinases which are upregulated in response to camptothecin derivatives like topotecan^33^,^34^ and radiation^35^,^36^. Sustained activation of ERK1/2, JNK or p38 plays a crucial role in the regulation of apoptosis, cell cycle progression, growth and differentiation^37^,^38^. JNK and p38 induce apoptosis by phosphorylating pro-apoptotic BAD^39^ and inactivation of anti-apoptotic Bcl-2 and Bcl-xL proteins^40^.

**Figure 9:**
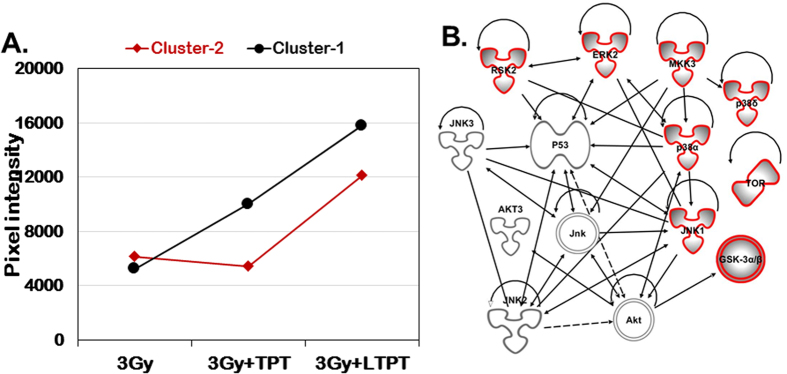
The trend of phophoprotein expression and signaling network. (**A**) Based on the pixel densities of the signal in each spot of the array corresponding to the respective phosphorylated protein, they were grouped in one of the two clusters. (**B**) Ingenuity pathway analysis shows the interaction of phosphorylated proteins that are significantly activated in irradiated TES incubated in LTPT for 10 days. Interestingly activation of these proteins is associated directly or indirectly with the phosphorylation of functional p53 (serine 46 residue) which is expected to be activated in endothelial cells only.

Based on the different phosphoprotein expression trends in TES in response to various treatment conditions, they were grouped into two clusters (Figs 8B and 9). In cluster-1 consisting of 6 phosphoproteins, the three treatment groups including radiation alone and free or LTPT with radiation exhibited a trend of increasing phosphorylation. However, the 8 phosphoproteins in cluster-2 appeared to be selectively upregulated in TES that were treated with LTPT and radiation (Fig. 9A). Of further interest was the phosphorylation of p53 at the serine 46 residue in cluster-1. Phosphorylation of the serine 46 residue is critical to triggering the p53 dependent apoptotic signaling cascade found to occur in normal cell types^41^. Apoptotic stress resulting in phosphorylation of ERK, p38, and JNK has also been associated with phosphorylation and stabilization of the p53 tumor suppressor protein^42^. However, within TES, only the 2H11 endothelial cells that have a functional p53 that will undergo activation in response to an apoptotic or stress signal. The 4T1 murine mammary carcinoma cells are p53 null and do not express the p53 protein^43^. The Ingenuity pathway analysis (IPA) elucidated that the phosphorylation/activation of these proteins is associated with p53 (serine 46 residue) phosphorylation and impacts the amplification of apoptotic signals (Fig. 9B). The data suggests that the metronomic effect of the combined treatment of liposomal TPT and radiation on endothelial cells may be a possible reason for efficient activation of ERK, JNK and p38 family of MAP kinases resulting in enhanced toxicity observed at day 10 in the TES.

## Discussion

The typical treatment options available for TNBC are a combination of therapies such as surgery, chemotherapy and radiation^44^. Chemotherapy followed by radiotherapy has been shown to significantly increase the survival outcomes in TNBC women after mastectomy^45^. Metronomic oral topotecan alone or in combination with pazopanib has been shown to prolong survival and reduce metastasis in gastrointestinal and gynecological cancers^5^,^46^,^47^. Its use in LDMC has been limited in other types of cancers due in part to its short half-life of 3.5 hours in the circulation^48^ and the need for frequent administration^47^,^49^. Continuous infusion via intravenous administration not being a logical option owing to the patients inconvenience, has met with limited success and not yet become a standard practice in the clinic^50^,^51^. Efficiently loaded nanoparticles not only stabilize the therapeutic agents and influence the drug’s biodistribution^52^, but may also facilitate sustained release at the vascularized tumor site^53^,^54^. This sustained release in turn provides low concentration of active drug at the tumor site over a prolonged period of time, thus simulating the action of localized metronomic therapy in cancer^16^.

The rational use of antineoplastic agents in pharmacotherapy requires preclinical studies to model the pharmacokinetic/pharmacodynamics (PK/PD) for predicting the effect and efficacy of drug dosing over time. While the alternate paradigm of metronomic therapy is gaining pace when compared to the traditional paradigm of cytotoxic chemotherapy based on the MTD, there is still no accepted definition of the ‘metronomic dose’. Majority of the clinical trials administering metronomic therapy have utilized an arbitrary dose range of 10–33% of the MTD as the representative metronomic dose^10^,^55^. Therefore, to maximize the antitumor response, metronomic approaches have yet to optimize the drug schedule and dosage. In this respect cost-effective *in vitro* models, that allow for controlled experimental manipulation, present a valuable predictive tool for optimization of dosing schedules before translation of the data to animals and clinical trials^56^. Advancing the efficacy of LDMC not only requires testing capable of determining the optimal dose but possible interaction of the formulation with other co-administered treatment modalities^12^,^57^. Current initiatives to address the specific issues of right dose, rhythm of drug administration and co-treatment modalities for LDMC utilize the novel approach of mathematical modeling of cancer growth^58^,^59^. A mathematical model based on the combined dynamics of the proliferating quiescent and necrotic tumor cells, endothelial cells and other normal cells along with the influence of the oxygen gradient and angiogenic factors was designed by Andre et. al to reproduce the expected efficacy of MTD and metronomic schedule for Temozolomide chemotherapy^59^. The usefulness of such mathematical models is in their versatility to adapt to several kinds of single or multi-agent therapeutic strategies. Multicellular tumor spheroids have served as ideal experimental model system for deriving the mathematical algorithms for cancer growth and associated therapy^60^,^61^,^62^. The 3D co-culture of tumor and endothelial cells in this study provides an improved system for the mathematical modeling of tumor and endothelial cell sensitivity to LDMC and determine the most effective drug/ therapeutic combination to translate to clinical settings.

In the current study we have developed a cost- effective tumor model for the screening and testing of metronomic and/or combinatorial therapeutic responses *in vitro*. The ability of the *in vitro* TES model to capture characteristics of the tumor microenvironment and continue to grow for several weeks in culture, unlike 2D monolayer cultures that need to be passaged in 3–4 days, enables analysis of the gradient of penetration/accumulation of drugs or nanoparticles over an extended period of time and provides a more predictive assessment of *in vivo* response. The 4T1 murine mammary carcinoma cells selected for co-culturing with the murine 2H11 endothelial cell line in this study exhibit growth and metastasis spread closely mimicking the human TNBC prognosis^63^. The 2H11 murine endothelial cell line isolated from the lymph nodes of adult C3H/He mice^64^ is very well characterized by the presence of features typical of normal endothelial cells and those of endothelial cells isolated directly from tumors^65^. These studies (Fig. 2) correlate with earlier reports that demonstrate that endothelial cells are more sensitive than tumor cells to TPT^32^ and radiation^66^,^67^. Recently redefined by Klement and Kamen, LDMC is the minimum biologically effective dose of a chemotherapeutic agent, which, when given at a continuous dosing regimen with no prolonged drug-free breaks leads to anti-tumor activity^68^ The antitumor activity of LDMC is due to increase in the sensitivity of proliferating endothelial cells in the tumor vasculature and inhibition of angiogenesis^10^,^69^. As demonstrated herein, sustained release of TPT from the liposomal formulation (LTPT) enhances TPT toxicity to the endothelial cells and provides similar efficacy in the TES model as metronomic therapy. Combined treatment with a single dose of LTPT and radiation also enhanced cytotoxicity and cell damage in the TES (Figs 4). Furthermore, semi-quantitative molecular studies using Phosho MAPK antibody arrays indicated that the enhanced TES cytotoxicity resulting from a combination of incubation with LTPT and radiation exposure is associated with the activation/ phosphorylation of p53 in endothelial cells as shown in Figs 8 and 9.

Efforts to evaluate complex combinations and novel cancer therapies have been challenging and require more representative yet cost-effective and reproducible *in vitro* models of human cancer. To evaluate metronomic therapy and radiation in preclinical *in vitro* models it is critical to have a more realistic representation of the tumor-endothelial cells, as both metronomic therapy and radiation function by affecting the tumor microenvironment. Further, the liposomal accumulation and drug release at the tumor site are extremely important for an understanding of the overall drug efficiency. Traditionally this information is obtained from *in vivo* studies in animal models. *In vitro* tumor models that can incorporate the principles of pharmacokinetics and pharmacodynamics (PK/PD) nanoparticle drug delivery to optimize therapeutic response may be considered the method of choice for optimizing the drug design prior to initiating expensive and resource-intensive experiments in animals^56^,^70^,^71^. The current studies demonstrate that a 3D TES model provides such representation of the tumor microenvironment by illustrating enhanced penetration and efficacy of liposomal formulations of TPT alone and in conjunction with a clinically-relevant dose of radiation.

## Materials and Methods

### Materials

Topotecan hydrochloride was purchased from AK Scientific (Union City, CA). Powders of 1, 2-distearoyl-sn-glycero-3-phosphatidylcholine (DSPC), >99%, and 1,2-distearoyl-sn-glycero-3-phosphoethanolamine-N-[methoxy(polyethyleneglycol)-2000] (ammonium salt) (m-PEG DSPE), >99% purity, were purchased from Avanti Polar Lipids (Alabaster, AL). Nuclepore® polycarbonate membranes (0.1 μm), Dowex® 50WX8-200 resin in the H^+^ form, Sephadex® G-25 PD-10 columns, solvents, and buffer salts were purchased from Fisher Scientific (Florence, KY). All solvents were HPLC grade. MTT (3-(4,5-dimethylthiazol-2-yl)-2,5-diphenyltetrazolium bromide) was purchased from Sigma-Aldrich (St. Louis, MO), and cell culture media was obtained from GIBCO Life Technologies (Carlsbad, CA).

### Actively-loaded Liposomal TPT Formulations

Large unilamellar liposomes containing 0.3 M ammonium besylate solutions were prepared as previously reported^72^,^73^. Briefly, DSPC and m-PEG-DSPE (95:5 molar ratio) were dissolved in chloroform. A thin lipid film was generated after evaporating CHCl_3_ under N_2_ and fully dried under vacuum at 35 °C for 6 hours. Dried films were hydrated with 0.3 M ammonium besylate solutions made via ion exchange using Dowex® 50WX8–200 as reported previously^24^. The resulting suspensions were vortexed at 60 °C, then extruded through two, stacked 0.1 μm polycarbonate membranes 10 times at 40 psig and 60 °C to yield ammonium besylate liposomes (ABLs) with a particle size (determined by dynamic light scattering) of 99.5 ± 3.5 nm and a polydispersity index of 0.13 ± 0.08 nm. Active loading of TPT into ABLs was performed by generating a low intravesicular pH via an ammonia gradient^7^. Extravesicular ammonia was removed to establish the gradient by passing 0.6 mL of ABLs through a Sephadex® G-25 size exclusion column pre-equilibrated with pH 5.5 100 mM 2-(N-morpholino)ethanesulfonic acid (MES) buffer. The first 2.5 mL fraction eluted from the column was discarded. The next 2.5 mL containing ABLs suspended in MES buffer was collected and 1.5 mL of the eluted suspension was mixed with an equal volume of TPT dissolved in the same pH 5.5 MES buffer to achieve a total extravesicular TPT concentration of 100 μM and a lipid concentration of 1.86 mg/mL. TPT active loading was performed for 1 hour at 60 °C with this suspension and achieved a loading efficiency of 72%. The resulting LTPT formulation was used for release studies after passing through a Sephadex-G25 column equilibrated with pH 7.4 phosphate buffer saline (PBS) or stored at 4 °C until needed.

### Liposomal Characterization

Liposome particle size was analyzed by dynamic light scattering with the aid of a Beckman Delsa™ Nano C Particle Sizer (Brea, CA) as reported previously^72^. TPT suspensions collected during active loading or release studies were diluted with acidified methanol (0.001 N HCl) and stored at −20 °C until analyses. Acidification converts TPT to its lactone form, which was later analyzed by HPLC^72^. Active drug loading and lipid composition of the liposomal formulations were determined as previously described^74^,^75^. Briefly, TPT lactone concentration was determined by HPLC using a Waters Symmetry C18 column (4.6 × 150 mm, 5 μm) and a mobile phase (16% acetonitrile:84% (v/v) of 5% (pH 5.5) triethylamine acetate buffer) at a flow rate of 1 mL/min. A fluorescence detector (M474) operating at excitation and emission wavelengths of 380 and 560 nm was used to monitor the lactone form of TPT, which exhibited a retention time of 4 min. A linear response was observed for TPT standards over the concentration range of 20–200 nM in acidified methanol. For lipid content determination, aliquots of liposome suspensions (100 – 250 μL) were dried at room temperature under N_2_, then dissolved in chilled solvent (80% chloroform:19.5% methanol:0.5%(v/v) NH_4_OH). Lipid samples were analyzed using a Waters Alliance 2695 separation module and an Allsphere (Alltech Associates, Inc., Deerfield, IL) silica column (4 × 150 mm, 5 μm) and guard column (20 × 4.0 mm, 5 μm) and a mobile phase (80% methanol:19.5% water:0.5% (v/v) NH_4_OH) at a flow rate of 1 mL/min. DSPC was quantified with this HPLC method using an evaporative light scattering detector (ELSD, Sedere, Inc., Lawrenceville, NJ) operated at 40 psig and 40 °C. DSPC standards (0.05 – 0.3 mg/mL) were dissolved in the same solvent as liposome samples. Plots of the logarithms of standard peak areas versus the logarithms of concentration were linear over this concentration range.

### *In-vitro* release kinetics of liposomal TPT in PBS or culture medium at pH 7.4

A previously developed fluorescence method capable of determining the release kinetics of LTPT in PBS and plasma was used to monitor LTPT release in PBS and cell culture media in this study^24^. Briefly, 0.5 mL of LTPT suspension was passed through a Sephadex® G-25 column equilibrated with PBS or cell culture medium to remove unentrapped TPT resulting in suspensions of TPT-loaded liposomes in the release media of interest. The first 2.5 mL eluted from the column was discarded and the next 2.5 mL containing LTPT was collected and diluted four fold with PBS or cell culture medium. TPT release was monitored at 37 °C using the red shift in TPT’s fluorescence excitation spectra that occurs as it is released^72^. Excitation spectra (290–500 nm) were collected at an emission wavelength of 550 nm using a FluoroMax-3, Jobin Yvon Inc (Edison, NJ) operated at 37 °C, 0.5 s integration time, and 1.5 nm slit width. Free TPT concentration was determined by comparing the fluorescence intensity (excitation of 410 nm) of samples to TPT standards (0.2 μM–5 μM) in PBS or culture medium as described previously^76^. A 100 μL aliquot of liposomal suspension was also dissolved in acidified Me OH and analyzed by HPLC to determine the total TPT suspension concentration at the beginning of each release study.

### Cell lines and Culture

4T1 is a highly metastatic murine mammary epithelial cell line, representative of the human triple negative breast cancer phenotype that does not express estrogen, progesterone and Her2 receptor^77^. Both 4T1 and green fluorescent protein expressing 4T1 (GFP-4T1) cell lines are used in this study. The 2H11 cell line validated as a tumor-like endothelial cell line by Walter-Yohrling *et al*.^65^ has attributes of both normal endothelial cells^28^ as well as endothelial cells directly isolated from tumors^78^. Tumor and endothelial cells were cultured in high-glucose Dulbecco’s modified Eagle’s medium supplemented with 5% fetal bovine serum, sodium pyruvate, non-essential amino acids, and 1% penicillin-streptomycin (Invitrogen, Carlsbad, CA) at 37 °C in a humidified atmosphere with 5% CO_2_.

### X-Ray Irradiation

High energy radiation (X-Ray) exposure was given using the Varian TrueBeam System (Palo Alto, CA) at the Department of Radiation Medicine at the University of Kentucky. The instrument was set at a radiation dose rate of 1.018 ± 0.10 Gy/min at 150 kV and 6.6 mA. The standard radiation dose in all experiments was 3 Gy.

### MTT assay

Cells were seeded at a density of 2 × 10^3^ cells/well in 96-well plates and incubated at 37 °C for 24 hours. Cells were treated with nine different concentrations of TPT (0.075–5 μM) with and without radiation exposure. The radiation was applied 6 hours after TPT treatment. Seventy-two hours later, 50 μL of MTT (5 mg/mL in PBS) was added to each well and incubated at 37 °C for 4 hours followed by dimethylsulfoxide (DMSO) solubilization of the cells. The dissolved chromogen in DMSO was measured at an absorbance wavelength of 540 nm using a Spectramax microplate reader (Molecular Devices LLC, Sunnyvale, CA) at 37 °C. The percentage of viable cells was determined by comparison with untreated controls. The median inhibitory concentration or IC50 (i.e., 50% reduction in cell viability) was determined for both tumor and endothelial cell lines.

### Clonogenic assay

One to five hundred cells were plated in 6-well culture plates and then treated with 1/2 and 1/4 the IC50 dose of TPT in triplicate. Cells were irradiated with 3 Gy of radiation 6 hours after drug treatment. After radiation exposure, plates were incubated at 37 °C and 5% CO_2_ for 10–12 days until large colonies (>1 mm) formed. The colonies were fixed and stained with 0.5% crystal violet (Sigma Aldrich, St. Louis, MO) in 50% methanol/water. Colonies were counted using ImageJ software and confirmed by manual counting.

Plating efficiency (PE) and survival fraction (SF) were calculated using following equations^79^:
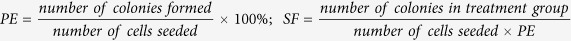


Survival fraction was determined for both tumor and endothelial cell lines after TPT treatment either in conjunction with or without radiation exposure. Survival fractions were calculated from n = 3 samples for each experiment.

### 3D “Hanging drop” co-cultures

Tumor-endothelial cell spheroids (TES) were generated by co-culturing 4T1 tumor cells and 2H11 endothelial cells in “hanging drops” of medium (Dulbecco modified Eagle medium with 10% fetal bovine serum and antibiotic mix) as previously described^28^. A single cell suspension of 4T1 cells and 2H11 cells (3000 cells/20 μL) was dispensed on the inside of the lid of each well of a 48-well cell culture plate, Greiner CellStar (Kaysville, UT). Spheroids were grown as hanging drops for 10 days and transferred to Greiner repellent plates when required for assessing treatment response. Spheroids composed of only GFP-4T1 cells were made with the same protocol.

### Toxicity evaluation of Liposomal TPT in TES

Randomly selected equal-sized TES were treated with free TPT or the nanoliposomal suspension (LTPT). The selected drug treatment groups were irradiated at 3 Gy after 6 hours. Drug containing media was replaced after 24 hours. Images for representation and quantitation of cell death, fluorescence and structural changes in the TES were obtained at day 10 using an Olympus FV1000 laser scanning confocal microscope (Olympus America Inc. Melville, NY) and a Typhoon FLA 7000 (GE Healthcare, Piscataway, NJ). TES from all treatment groups were imaged for green fluorescent protein (GFP) in 4T1 cells and stained for propidium iodide (PI) to quantify the abundance of dead cells.

### Scanning electron microscopy

4T1 tumor cell and 4T1-2H11 tumor-endothelial cell spheroids grown in hanging drops for 10 days were transferred to Formvar/Carbon grids (Ted Pella Inc, Redding, CA). The spheroid containing grids were imaged with an S-3200-N Hitachi scanning electron microscope at an accelerating voltage of 16 kV to observe the spheroid compactness or integrity and extracellular matrix formation.

### Immunohistochemistry

Spheroids were harvested and frozen in Tissue-Tek® O.C.T., (Miles USA, Inc. Elkhart, IN). Eight μm cryostat sections were obtained with a Leica CM1950 cryotome and transferred on to Superfrost™ Plus slides (Fischer Scientific, Boston, MA) for immunostaining. Slides were incubated in polyclonal rabbit anti-human fibronectin (Q0149, Dako) and collagen Type IV mouse monoclonal antibodies for 30 min at room temperature followed by labelling using a Dako Envision + kit (IR630) from Dako North America Inc. (Carpinteria, CA) according to the manufacturer’s instructions. Signal was visualized with 3,3′-Diaminobenzidine (DAB) followed by a light hematoxylin counterstain. Images were taken at 40X magnification using a Nikon Ti E upright microscope with a Cool SNAP HQ2 CCD camera (Tokyo, Japan) and processed with NIS-Elements basic research software.

### Hematoxylin and Eosin Staining and Imaging

Hematoxylin and eosin (H&E) staining was performed by staining the cryostat sections of TES with Harris hematoxylin (aluminum potassium sulfate, hematoxylin, absolute alcohol, mercuric oxide, and glacial acetic acid) followed by 1% acid alcohol and, subsequently, 1% eosin. Images of spheroids were obtained as described above.

### Microscopy and image analyses

TES fluorescence images were captured using a Olympus FV1000 laser scanning confocal microscope (Olympus America Inc. Melville, NY) equipped with 10X and 20X objectives (NA = 1.32). Two channels were acquired sequentially with the following excitation and emission parameters: 405 and 465–500 nm for accumulation of TPT or GFP expression in tumor cells and 543 and 555–615 nm for PI staining of dead cells. The monochromatic differential interference contrast (DIC) images provided assessment of TES morphology and size. Gains were adjusted to avoid saturation in pixel intensity. Each TES image consisted of 24 optical images separated by 5 μm along the z-axis captured at a 512 × 512 resolution and a speed of 10 μs/pixel for both channels. A DIC image was acquired after each Z-stack image for accurate sizing. All 24 optical slices were collapsed to generate a maximum (i.e. cumulative) intensity projection image with the FV10-ASW 1.7 image processing software. TPT/GFP or PI staining was quantified using an Olympus image analysis or ImageJ 1.47v (NIH, Bethesda, USA) software.

### Phospho MAPK Antibody Array

TES harvested 10 days post-treatment were homogenized using a 21-gauge syringe in lysis buffer as described earlier^28^. Insoluble material were removed by centrifugation (15 min, 12000 g). The protein concentration was determined using micro BCA protein assay kit (Thermo scientific Pierce, Rockford, IL). 125 μg of lysate was hybridized to a human phospho MAPK antibody array (R&D Systems Inc, Minneapolis, MN) as instructed.

### Pathway Analysis

The Ingenuity Pathway Analysis Tool was used to identify gene networks and examine the functional associations between differentially expressed proteins in the MAPK arrays obtained from incubation with lysates of TES following treatment (www.ingenuity.com).

### Statistical analyses

Data are expressed as mean ± SD of at least three different experiments unless otherwise mentioned. Cell survival, sizing, and PI/GFP data for TES were represented as means (N = 4–12 TES per group) ± SD. Statistical analyses were performed using Graphpad Prism software version 5.04 (GraphPad.com). Two tailed unpaired t-tests with Welch’s correction was used to determine statistical significance. The level of significance was accepted at *p* < 0.05. For analysis of data from the Phospho MAPK antibody arrays, K-means clustering in conjunction with correlation metric was used to partition the markers into two clusters based on their expression profile across the three conditions corresponding to the following treatments: radiation (3 Gy), 3 Gy + TPT and 3 Gy + LTPT.

## Additional Information

**How to cite this article**: Jyoti, A. *et al*. An *in vitro* assessment of liposomal topotecan simulating metronomic chemotherapy in combination with radiation in tumor-endothelial spheroids. *Sci. Rep*. **5**, 15236; doi: 10.1038/srep15236 (2015).

## Supplementary Material

Supplementary Information
