# Supplementary material for: An in vitro assessment of liposomal topotecan simulating metronomic chemotherapy in combination with radiation in tumor-endothelial spheroids
Source: Sci Rep. 2015 Oct 15;5:15236. doi: 10.1038/srep15236 (PMC4606561; doi:10.1038/srep15236)
Supplement: Supplementary Information [file srep15236-s1.pdf]

## SUPPLEMENTARY INFORMATION

# An *in vitro* assessment of liposomal topotecan simulating metronomic chemotherapy in combination with radiation in tumor-endothelial spheroids

Amar Jyoti[1], Kyle D Fugit[1], Pallavi Sethi[1], Ronald C. McGarry[2], Bradley D. Anderson[1], Meenakshi Upreti[1]*.

[1]Department of Pharmaceutical Sciences, University of Kentucky, Lexington, KY; [2]Department of Radiation Medicine, University of Kentucky Chandler Hospital, Lexington, KY

***Corresponding Author:** Meenakshi Upreti

555, Biopharmaceutical Complex

College of Pharmacy, University of Kentucky,

789 S. Limestone Street,

Lexington, KY 40536-0596, USA

Tel.: +1 859-218-1041

E-mail address: m.upreti@uky.edu

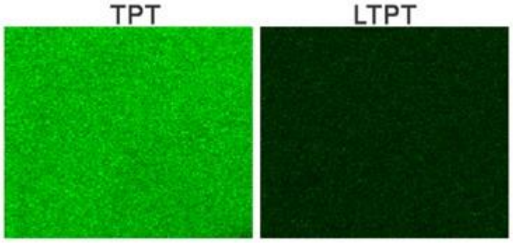


**Fig-S1.** Confocal images showing the intensities of TPT fluorescence (5 μM) when TPT either present as free or entrapped within liposomes (LTPT) at physiological pH7.4. Entrapped TPT exhibits extremely low fluorescence compared to free TPT in the TES culture medium.
